# FERAL: A Video-Understanding System for Direct Video-to-Behavior Mapping

**DOI:** 10.1101/2025.11.16.688666

**Published:** 2025-11-17

**Authors:** Peter Skovorodnikov, Janet Zhao, Friederike Buck, Tomas Kay, Dominic D. Frank, Benjamin Koger, Blair R. Costelloe, Iain D. Couzin, Jacopo Razzauti

**Affiliations:** 1 Data Science Platform, The Rockefeller University, New York, NY, USA; 2 Laboratory of Social Evolution and Behavior, The Rockefeller University, New York, NY, USA; 3 Lulu and Anthony Wang Laboratory of Neural Circuits and Behavior, The Rockefeller University, New York, NY, USA; 4 School of Computing, University of Wyoming, Laramie, WY, USA; 5 Department of Zoology and Physiology, University of Wyoming, Laramie, WY, USA; 6 Department of Collective Behaviour, Max Planck Institute of Animal Behavior, Konstanz, Germany; 7 Department of Biology, University of Konstanz, Konstanz, Germany; 8 Centre for the Advanced Study of Collective Behaviour, University of Konstanz, Germany; 9 Laboratory of Neurogenetics and Behavior, The Rockefeller University, New York, NY, USA; 10 The Price Family Center for the Social Brain, The Rockefeller University, New York, NY, USA; 11 Howard Hughes Medical Institute, New York, NY, USA

## Abstract

Animal behavior unfolds continuously in time, yet quantitative analyses often require segmenting it into discrete, interpretable states. Although manual annotation can achieve this, it remains slow, subjective, and difficult to scale. Most automated pipelines use tracked body parts to infer actions, but are limited by tracking quality, and discard much of the visual information contained in raw videos. Here we present FERAL (Feature Extraction for Recognition of Animal Locomotion), a supervised video-understanding toolkit that bridges this gap by mapping raw video directly to frame-level behavioral labels, bypassing the need for pose estimation. Across benchmarks, FERAL outperforms state-of-the-art pose- and video-based baselines: on a benchmarking dataset of mouse social interaction, it surpasses Google’s Videoprism using just a quarter of the training data. FERAL generalizes across species, recording conditions, and levels of behavioral organization: from single-animal locomotion to complex social interactions and emergent collective dynamics. Released as a user-friendly, open-source package, FERAL overcomes the challenges of traditional approaches, integrates easily with existing analysis pipelines, and can be deployed locally or on cloud servers with a few clicks. By mapping raw video directly to annotated behavior, FERAL lowers the barrier to scalable, cross-species behavioral quantification and broadens the range of behavioral analyses possible in both the lab and the wild.

## Introduction

Quantifying animal behavior remains a central challenge in biology, linking neural activity, evolution, and ecology through the study of discrete, observable actions [1–4]. Since the earliest days of ethology, researchers have identified these actions through naturalistic observation and manual annotation [5–9], establishing the foundation of modern behavioral science [3, 10–12]. Such analyses have been essential across disciplines, informing research in ecology [13], neuroscience [14], disease modeling, and drug discovery.

**Figure 1:**
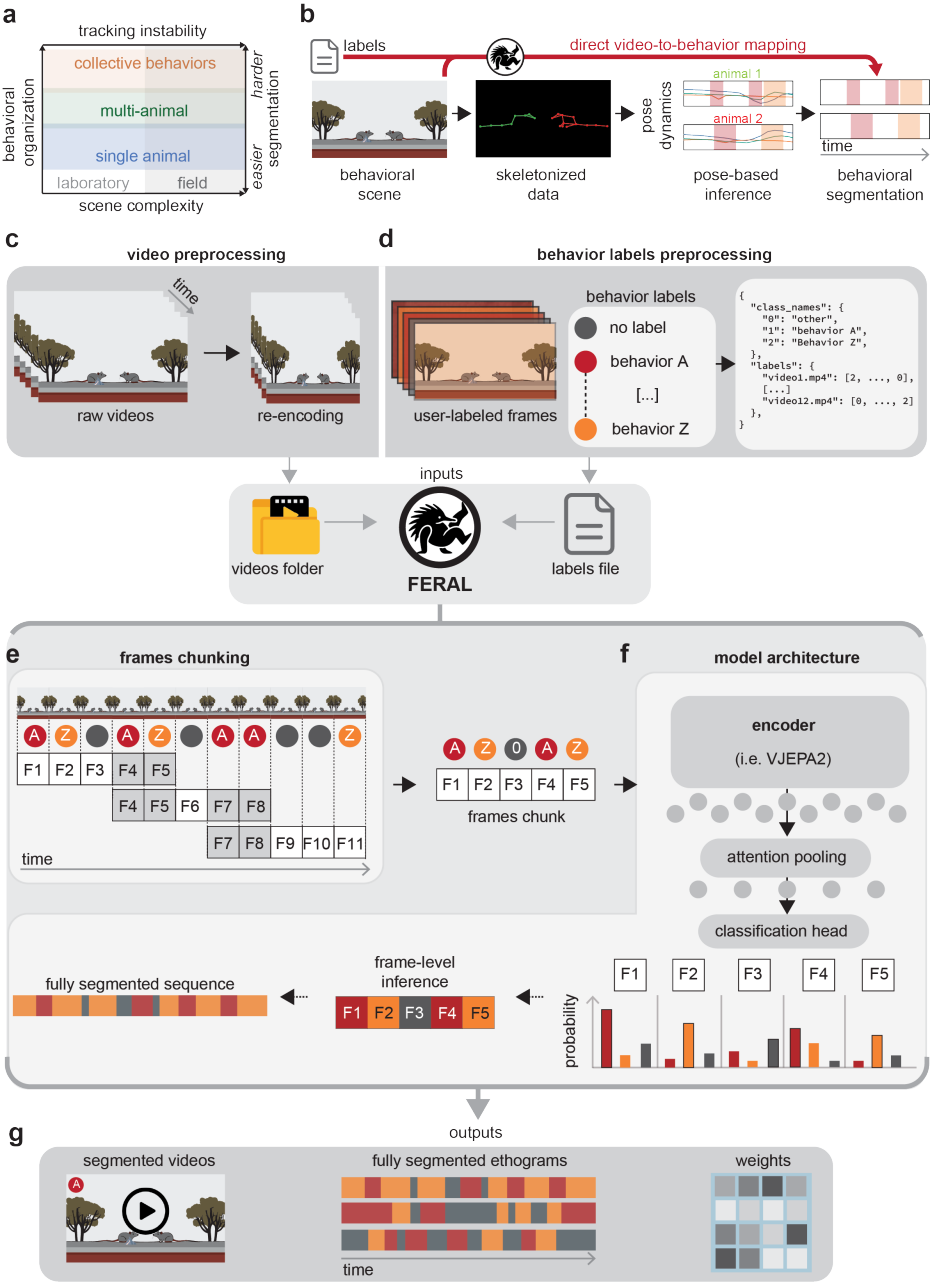
Overview of the FERAL workflow **(a)** Conceptual space of behavioral analysis: increasing scene complexity (left to right) and level of organization (bottom to top) challenge tracking and pose-based pipelines for behavior segmentation. **(b)** Direct video-to-behavior mapping: FERAL learns from raw scenes and expert labels, bypassing skeletonized pose to produce frame-level segmentations. **(c)** *Video preprocessing*: raw videos are resized and re-encoded into standardized, seekable inputs. **(d)** *Behavior labels preprocessing*: user annotations are converted to a unified frame-aligned JSON schema (class names and per-frame label arrays). **(e)** *Frames chunking*: videos are divided into overlapping temporal segments. **(f)** *Model training*: chunks are embedded by a pretrained video encoder (i.e., V-JEPA2); attention pooling aggregates spatiotemporal features followed by a lightweight head. Overlapping predictions are ensembled to produce stable frame-level probabilities. **(g)** *Outputs*: segmented videos, ethograms, and model weights for reuse or further fine-tuning.

Manual annotation remains the most versatile method for quantifying behavior, adapting to scene complexities that range from controlled arenas to field recordings. However, it is labor-intensive, subjective, and the main bottleneck in scaling behavioral research [15–17]. Advances in computer vision have partially mitigated this limitation through markerless tracking tools such as DeepLabCut [18], SLEAP [19], and LightningPose [20], which estimate the positions of animals and their body parts over time [21, 22]. These tools have transformed behavioral analysis in laboratory settings, but still require tightly controlled imaging conditions. In more naturalistic or noisy environments, they often fail, forcing researchers to simplify behavioral assays (e.g., by tethering animals or designing task-specific arenas), rely on manual annotation, or to abandon complex analyses altogether (Figure 1a–b). Importantly, these methods answer where animals (and their body parts) are, but not what actions they are performing.

Automatically determining the actions animals are engaged in remains a greater challenge. Current computational pipelines for behavioral segmentation depend almost entirely on pose-based outputs, transforming keypoint trajectories into discrete behavioral states [2, 23–28]. Analyses based on these intermediate, skeletonized representations are limited by tracking quality and feasibility, and discard rich contextual information that is often essential for accurate behavioral interpretation. As the level of behavioral organization increases, such as in social or collective dynamics, these pipelines become increasingly complex, requiring multi-animal tracking and extensive post-tracking curation (Figure 1a). In many cases, the visual cues that define a behavior are well established, and researchers primarily need a scalable, automated way to recognize them across large datasets.

To bridge this gap, we developed FERAL (Feature Extraction for Recognition of Animal Locomotion), a supervised video-understanding system that learns behavior directly from raw video frames, bypassing the need for pose trajectories while preserving full temporal and visual information (Figure 1b). This approach is particularly advantageous when pose estimation is unreliable or unnecessary for the analysis at hand. FERAL can operate either alongside or independently of existing pose-based frameworks, providing an end-to-end solution for context-rich behavioral segmentation.

Leveraging a pretrained video foundation model [29], FERAL integrates motion and visual cues within a unified architecture. Trained directly on videos aligned with expert annotations, it detects discrete actions and generates interpretable behavioral sequences (i.e., ethograms) that reliably capture discrete animal behavior.

On benchmarking datasets, FERAL consistently outperforms state-of-the-art pose- and video-based baselines. It generalizes robustly across seven datasets spanning diverse species, recording modalities (from controlled laboratory conditions to field and aerial footages), and levels of behavioral organization: from single-animal locomotion in *C. elegans* to social interactions and emergent collective dynamics in rodents, ants, and primates. These results demonstrate that FERAL maintains high performance even in scenarios where traditional pipelines typically struggle. For instance, FERAL makes analysis of behaviors involving extensive interaction of animals either with each other or with their environments much more feasible.

Designed for ease of use, FERAL unifies preprocessing, training, and inference within a modular, user-friendly workflow requiring no coding or machine learning expertise. With only a few lines of Python it can be run locally or on cloud platforms such as Google Colab, providing a scalable, context-aware foundation for modern behavioral science.

## Results

### Direct video-to-behavior mapping with FERAL

FERAL provides a user-friendly, end-to-end workflow that takes raw videos and behavioral annotations as inputs, fine-tunes an open-source video-understanding model, and outputs segmented behavioral sequences. To ensure reproducibility across laboratories and recording setups, the pipeline standardizes both inputs (videos and labels) into a unified format.

The first stage is *video preprocessing*. Because video-understanding models operate on relatively low-resolution inputs (e.g., 512×512 pixels), input videos must be resized and re-encoded into seekable formats to enable efficient frame sampling [29, 30]. FERAL includes a cross-platform function that automates this process, producing standardized inputs regardless of the original recording format (Figure 1c).

The second stage, *label preparation*, converts behavioral annotations from diverse software (e.g., BORIS [31], EthoVision [32]) into a consistent schema [31, 33]. The annotations provided by the user, often timestamp-based, are converted into JSON files that map every video frame to a categorical behavioral label (Figure 1d). In practice, users need only a folder of videos and a corresponding label file to initiate training and inference.

Because full-length recordings cannot be processed in a single pass, FERAL divides each video into overlapping temporal chunks that are independently processed and corresponding predictions are subsequently ensembled (Figure 1e). This design minimizes misclassification at behavioral boundaries and maintains temporal continuity by ensuring that each short behavior is fully captured within at least one chunk.

After evaluating several potential backbones, we adopted V-JEPA2 [29], a recent foundation model introduced by Meta FAIR (see Methods). Trained self-supervised on over one million hours of unlabeled video, V-JEPA2 learns rich spatiotemporal embeddings through a masked-prediction objective, producing a robust representation of input videos. Within FERAL, we fine-tune only a subset of its transformer layers using comparatively small, annotated datasets (i.e., the user-provided videos and labels). This aligns pretrained representations with the specific demands of behavioral segmentation rather than retraining the model from scratch [34] (Figure 1f).

To generate frame-level predictions, FERAL extends the encoder with an attention-based pooling module and a classification head. Each video chunk is represented as a sequence of spatiotemporal tokens that are integrated by a transformer, then compressed via attention pooling into temporally aligned embeddings. These embeddings are normalized and linearly projected into class logits, producing frame-wise probabilities that are ensembled across overlapping segments.

The resulting outputs are interpretable ethograms that describe what actions animals are performing at each frame. These can be visualized directly or integrated into downstream analyses. Model weights are automatically saved, allowing users to reuse or fine-tune trained networks on new datasets without retraining from scratch (Figure 1g).

Together, these design choices make FERAL both powerful and accessible, establishing a practical and reproducible framework for direct video-to-behavior mapping.

### FERAL outperforms state-of-the-art methods across benchmarks

**Figure 2:**
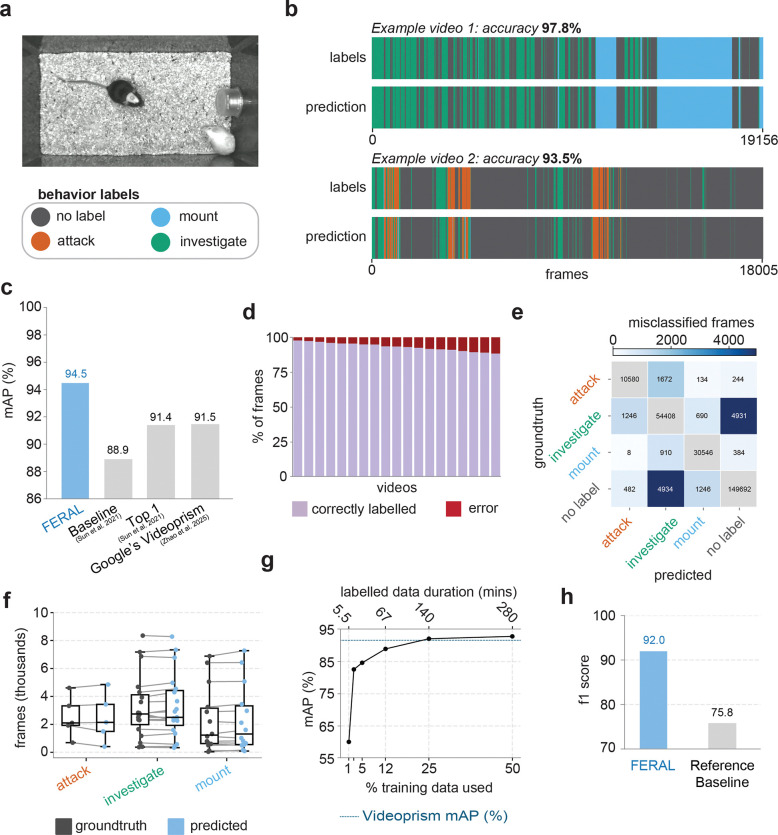
FERAL outperforms state-of-the-art baselines across benchmarks. **(a)** Example frame and corresponding behavioral labels from the CalMS21 dataset. **(b)** Representative ethograms comparing FERAL predictions (bottom) to expert annotations (top). **(c)** Mean average precision across models on the CalMS21 dataset. **(d)** Fraction of correctly (lavender) and incorrectly (red) classified frames per video. **(e)** Confusion matrix of misclassified frames. **(f)** Comparison between groundtruth and predicted frame counts for each behavioral category across videos. **(g)** Data-efficiency analysis showing mean average precision (mAP) as a function of the percentage of training data used (1%, 2.5%, 5%, 12%, 25%, 50%). **(h)** F1 score comparison between FERAL and the reference baseline on the MaBE dataset.

To evaluate FERAL’s precision and robustness relative to existing approaches, we benchmarked its performance on two established datasets that provide raw videos aligned with frame-level behavioral annotations. The first, the Caltech Mouse Social Interactions (CalMS21) dataset [35], contains recordings of freely behaving mice engaged in resident–intruder assays, paired with both tracked poses and expert behavioral labels (Figure 2a). The second, MaBE (Multi-Agent Behavior Benchmark) [36], is a large-scale, multi-species dataset spanning mice, beetles, ants, and flies, each annotated across diverse behavioral categories. Together, these datasets test FERAL’s core capability: direct video-to-behavior mapping without reliance on pose trajectories or bounding-box detections. Because only the beetle subset of MaBE provides both raw video and synchronized expert annotations, our evaluation focused on this subset.

Originally introduced as a community challenge for the classification of social behavior, the CalMS21 dataset included multiple baseline models and public submissions. We compared FERAL against a suite of alternative pose- and video-based approaches using mean average precision (mAP), the official metric of the original challenge. The strongest released baseline, in addition to the labeled behavioral dataset, employed self-supervised pretraining on large sets of unlabeled pose trajectories [35]. We also report the Competition Top-1 entry from the official leaderboard and results from Google’s VideoPrism paper [37]. This closed-source video-understanding model was fine-tuned on CalMS21 by freezing its backbone and training only the attention-pooling and classification heads.

FERAL achieved the highest overall performance on CalMS21, reaching 94.5%, outperforming the strongest baseline (88.9%), competition Top-1 (91.4%), and VideoPrism (91.5%) models (Figure 2b–c, **Supplementary Video 1**). FERAL also maintained consistent performance across videos, correctly classifying the majority of the frames within each sequence (Figure 2d). Confusion-matrix analysis (Figure 2e) showed that residual errors were mostly confined to confusions between “no label” and “investigate” categories, indicating occasional uncertainty in distinguishing background periods from subtle actions. Quantitative comparison of predicted and annotated total behavior durations per video (Figure 2f) confirmed high agreement across all behavioral categories, demonstrating that FERAL accurately preserved both the temporal structure and balance between classes of the annotated behavioral data.

To assess how much labeled data FERAL requires to achieve strong performance, we progressively subsampled the training set of CalMS21 (Figure 2g). FERAL achieved a mAP of 92.8 using only 50% of the available training data and 92.1 with 25%, already surpassing both the VideoPrism and Competition Top-1 models trained on the full dataset. Even when trained with only 12% of the data, performance remained high (89.0%), and robust mAP was maintained down to 5% (84.6%) and 2.5% (82.6%). At the lowest sampling level (1%), FERAL still achieved 60.0%, demonstrating strong data efficiency. These results highlight that FERAL achieves high and stable performance with minimal annotation effort.

For the beetle subset of *MaBE*, we evaluated FERAL’s frame-level predictions against expert annotations using the macro-averaged F1 score, following the evaluation protocol of the original publication. The top-performing submission in the *MaBE* challenge achieved a score of 0.758, which we adopt here as the reference baseline [36]. FERAL exceeded this performance by a wide margin (0.92) (Figure 2h). Note that in the competition, participants did not have access to labeled data for the target behaviors during training, so it is expected that FERAL, which is trained directly on the target-behavior labels, achieves higher performance.

### FERAL captures the temporal structure and visual appearance of behaviors

To assess FERAL’s ability to generalize across species and recording modalities, we applied it to two datasets, both acquired in laboratory conditions, representing distinct levels of behavioral organization and scene complexity.

#### Single-animal behavior in *C. elegans*.

**Figure 3:**
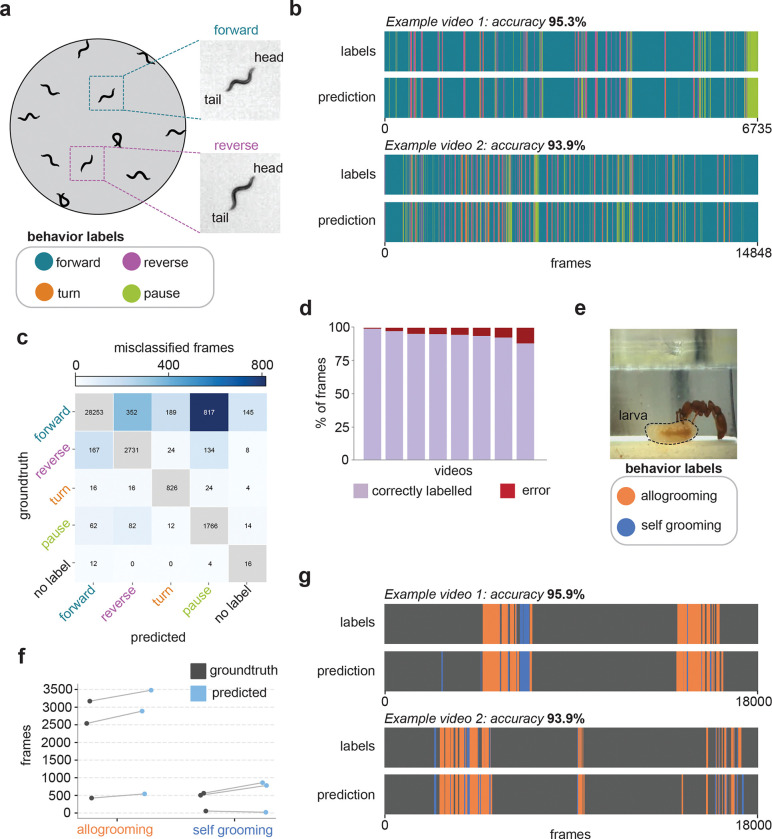
FERAL captures the temporal and visual appearance of behaviors. **(a)** Example frames from the *C. elegans* dataset showing forward and reverse locomotion states. **(b)** Representative ethograms comparing groundtruth annotations (top) and FERAL predictions (bottom) for *C. elegans* locomotory states. **(c)** Confusion matrix of misclassified frames across the four locomotor states (*forward*, *reverse*, *turn*, *pause*). **(d)** Fraction of correctly (lavender) and incorrectly (red) classified frames per video for *C. elegans*. **(e)** Example frame from recordings of dyadic interactions between an adult and larval clonal raider ant (*Ooceraea biroi*). **(f)** Quantitative comparison of groundtruth and predicted frame counts for each grooming category. **(g)** Representative ethograms from *O. biroi* videos comparing groundtruth and FERAL predictions for self-grooming and allogrooming events.

This dataset is comprised of recordings of freely moving *Caenorhabditis elegans* performing four canonical locomotor behaviors: forward crawling, reverse crawling, turning, and pausing (Figure 3a) [38, 39]. Unlike other datasets used in this study, these behavioral labels were not manually annotated; instead, each frame was automatically assigned to a locomotory state using a rule-based heuristic pipeline adapted from prior work [38]. In this pipeline, worms were segmented from the background, centroid positions were extracted, head–tail orientation was inferred from midline dynamics, and locomotor state was determined using speed thresholds, direction of motion, and self-intersection criteria (see Methods).

For each video, the centroid of the worm was continuously tracked, and a cropped image window centered on the animal was extracted to maintain consistent framing. FERAL leverages temporal context by integrating information from preceding and subsequent frames to classify each moment in time. As a result, it accurately segmented all four behavioral states and correctly distinguished between forward and reverse locomotion. These two locomotory states appear nearly identical in single frames but become separable through their temporal dynamics (Figure 3b, **Supplementary Video 2**).

Confusion-matrix analysis (Figure 3c) showed that the majority of errors were confined to transitions between kinematically adjacent states, such as “forward” versus “pause” or “forward” versus “reverse.” Across videos, FERAL maintained very high frame-level accuracy (Figure 3d). Beyond its strong performance, this analysis illustrates how FERAL can be seamlessly integrated into existing behavioral pipelines as a post-tracking module, complementing traditional centroid-based approaches by directly mapping cropped video segments to behaviors.

#### Dyadic interactions in *Ooceraea biroi*.

We next tested FERAL on a dataset capturing adult–larva interactions in the clonal raider ant *Ooceraea biroi*, which included expert annotations (J.Z.) for self-grooming and allogrooming events (Figure 3e). Self-grooming involves individuals cleaning their own body, while allogrooming targets another colony member which, in this case, is a larva. Accurate classification thus requires recognizing not only motion patterns but also the spatial relationship between the adult and the larva. This poses a major challenge for pose-based pipelines: (i) pose estimation is unreliable under frequent occlusions; and (ii) modeling these social interactions using skeletonized data requires integrating information from multiple individuals.

FERAL bypasses the need for pose estimation and pose-based segmentation, thus avoiding these challenges altogether. It reliably identified both self- and allogrooming events directly from raw videos (**Supplementary Video 3**), maintaining the overall temporal dynamics of each behavior across videos with high fidelity to the original expert annotations (Figure 3f–g).

Altogether, these results highlight FERAL’s ability to extract meaningful behaviors from spatiotemporal patterns that are difficult to access with single-frame or pose-based approaches, demonstrating its capacity to generalize from individual locomotion to social behaviors.

### FERAL generalizes to field recordings of wild animals

Given FERAL’s robust performance on datasets acquired in the laboratory, we next evaluated its generalization to field recordings, which typically exhibit higher scene complexity than laboratory videos.

#### Vigilance behavior in zebras.

**Figure 4:**
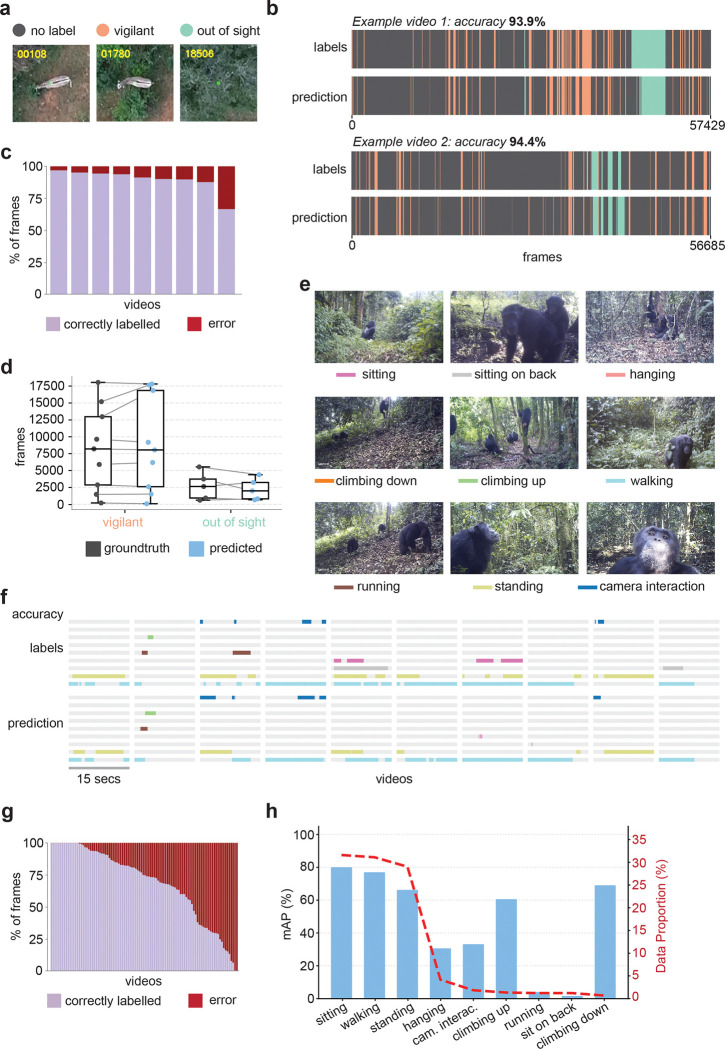
FERAL generalizes to field recordings of wild animals. **(a)** Representative drone frame from recordings of wild Grevy’s zebras (*Equus grevyi*) in Kenya. **(b)** Example ethograms comparing expert annotations (top) and FERAL predictions (bottom) for the zebras dataset. **(c)** Fraction of correctly (lavender) and incorrectly (red) classified frames per video for the zebras dataset. **(d)** Quantitative comparison of predicted and annotated vigilance and out-of-sight bout durations across videos for the zebras dataset. **(e)** Representative frames from the PanAf500 dataset [41], showing wild chimpanzees recorded by camera traps in forest environments, with one example frame for each annotated behavioral class. **(f)** Representative ethograms comparing expert labels (top) and FERAL predictions (bottom) across locomotor and postural behaviors for the PanAf500 dataset. **(g)** Fraction of correctly (lavender) and incorrectly (red) classified frames per video for the PanAf500 dataset. **(h)** Mean average precision (mAP) per behavioral class (bars) and labeled data proportion (red line) for each behavioral class for the PanAf500 dataset.

This dataset consisted of videos of wild Grevy’s (*Equus grevyi*) captured using a drone in Kenya. Free-ranging groups of zebras were filmed from a nadir (directly overhead) perspective. As with the *C. elegans* dataset, the centroid of each animal was continuously tracked following methods described in [40] and a cropped image window was centered on the animal to maintain consistent framing. Individual videos were annotated in BORIS by an expert (B.R.C.) to identify bouts of vigilance behavior, defined as the individual standing still with its head raised (Figure 4a). Due to the nadir perspective, the vertical head position of the zebras is challenging to detect, and previous attempts using pose estimation methods were unsuccessful at reliably identifying vigilance bouts.

FERAL accurately detected the onset and duration of vigilance periods directly from raw video (Figure 4b), closely matching expert annotations across most recordings. FERAL also accurately detected the frames in which the animal was out of sight. The fraction of correctly labeled frames was consistently high across all videos (Figure 4c). Quantitative comparison between predicted and annotated frame durations showed strong agreement for both *vigilant* and *out-of-sight* states (Figure 4d), indicating that FERAL successfully generalized to aerial perspectives and preserved temporal precision even under the natural variability of field conditions.

#### Chimpanzee and gorilla behavior in the wild.

To further assess FERAL’s generalization to complex, naturalistic scenes, we evaluated its performance on the *PanAf500* dataset [41], which contains camera-trap videos of wild chimpanzees (*Pan troglodytes*) and gorillas recorded across multiple African field sites as part of the Pan African Programme: The Cultured Chimpanzee. Each clip was manually annotated with fine-grained behavioral categories, including locomotor (e.g., walking, climbing, running), postural (e.g., standing, sitting, hanging), and interaction-related behaviors (Figure 4e). Multiple behavior labels can be assigned to the same frame, making this a multi-class, multi-species dataset that tests FERAL’s capacity to handle visual and behavioral complexity.

Despite substantial variation in lighting, vegetation, and visibility typical of camera-trap footage, FERAL accurately segmented common postural and locomotor behaviors such as *sitting*, *walking*, *standing*, and *climbing* (both up and down), achieving an overall mean average precision of 65.7%. The fraction of correctly labeled frames, where predictions for all behaviors are correct, varied widely across videos, possibly reflecting differences in scene complexity, visibility, and behavioral diversity (Figure 4f–g, **Supplementary Video 4**). While rare classes such as *running*, and *sitting on back* exhibited lower precision, visually distinctive yet infrequent behaviors like *climbing up* and *climbing down* were detected with high average precision despite their low representation in the labeled data (Figure 4h).

Together, these results demonstrate that FERAL’s performance on videos acquired in laboratory settings transfers robustly to field recordings spanning diverse species, habitats, and acquisition modalities. Its ability to extract meaningful behavioral structure directly from raw video highlights the model’s versatility and scalability for quantifying natural behavior in ecologically realistic contexts.

### FERAL identifies emergent collective behaviors directly from raw video

**Figure 5:**
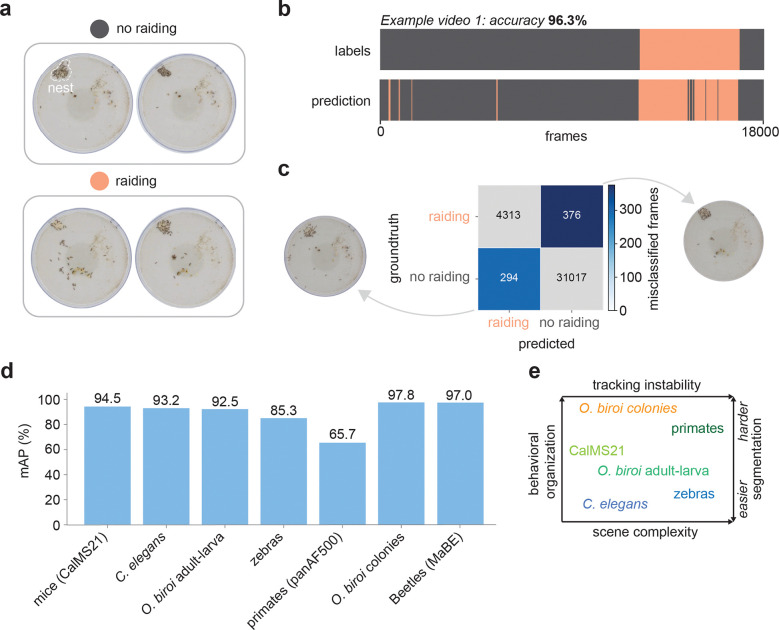
FERAL captures emergent collective behavior and achieves high performance across dataset. **(a)** Representative frames from recordings of clonal raider ant (*Ooceraea biroi*) colonies during non-raiding (top) and raiding (bottom) phases, with nests indicated by dashed outlines. **(b)** Example ethogram comparing groundtruth annotations (top) and FERAL predictions (bottom). **(c)** Confusion matrix showing correspondence between groundtruth and predicted frames across all videos. Representative frames showing visual appearance of misclassified frames. **(d)** mAP of FERAL across datasets tested in this study. **(e)** Summary of diversity in both behavioral organization and scene complexity across the dataset tested in this study.

To test whether FERAL generalizes from individual and dyadic interactions to collective dynamics, we applied it to recordings of clonal raider ant (*Ooceraea biroi*) colonies filmed continuously over several days (Figure 5a). In this species, foraging occurs as synchronized group raids [42]. Manual annotation of such raiding events was used as ground truth. Despite the high density of individuals, FERAL accurately detected the onset and duration of collective raids directly from raw video frames (Figure 5b). FERAL captured these events as emergent visual signatures without requiring individual tracking or explicit modeling of group structure. By contrast, conventional multi-animal tracking approaches would need to reconstruct trajectories for each individual [21, 43] and subsequently infer collective states through post-tracking analyses [42]. FERAL bypasses these steps, enabling scalable, tracking-free quantification of collective behavior.

Confusion-matrix analysis (Figure 5c) revealed that misclassified “raiding” frames were not random but systematically associated with moments of intense movement, when many ants had exited the nest and were exploring the arena. Conversely, most “no raiding” misclassifications occurred during phases of a raid when most ants were in the nest and overall activity declined. These patterns suggest that FERAL’s classifications are driven by emergent visual cues, such as colony-level motion and spatial distribution of individuals, demonstrating that the model learns the collective visual signature of raiding behavior directly from scene appearance.

By recognizing colony-level behaviors directly from video, FERAL extends the reach of direct video-to-behavior analysis to emergent social dynamics that arise from distributed coordination among many animals. This ability opens new possibilities for studying the neural, ecological, and evolutionary principles governing collective behavior across species and environments.

## Discussion

The quantitative study of animal behavior has advanced rapidly with the integration of computer vision and deep learning. Existing methods succeed at answering where animals and their body parts are over time, yet determining what actions animals are performing remains challenging, especially for complex environments. This limitation constrains discovery across ethology, neuroscience, and ecology, where understanding behavior and its structure is essential.

FERAL addresses this gap by reframing behavioral quantification as a video-understanding problem. Instead of relying on intermediate abstractions such as pose or trajectory, FERAL maps raw video directly to frame-level behavioral labels, capturing both the temporal and visual appearance of animal actions. It achieves high performance across datasets, detecting discrete actions with high precision on a wide range of scene complexities (Figure 5d–e). This generalization is enabled by its design. Its foundation-model backbone (e.g., V-JEPA2) allows robust fine-tuning even with limited behavioral annotations [29].

FERAL is an open-source, freely available toolkit that includes comprehensive documentation, tutorials, and benchmark datasets (www.getferal.ai), lowering the technical barrier for users across disciplines. Its modular architecture supports all stages of analysis, from preprocessing and training to inference, and can be deployed locally, on high performance clusters, or through cloud-based GPU services such as Google Colab and RunPod.

FERAL also integrates easily with existing pipelines. Although it focuses on frame-level segmentation and does not assign persistent identities, it can be paired with multi-animal tracking systems such as TRex [21], which produce animal-centered video segments suitable for direct inference. Together, these tools provide a unified solution to the two central questions of behavioral quantification: where animals are and what actions they are performing.

By combining strong performance with user-friendly design, FERAL establishes a robust foundation for behavioral segmentation across species and experimental paradigms. Behavior is central to many disciplines, ranging from neuroscience to ecology, and its quantitative analysis is a common point of entry to understand how biological phenomena work. FERAL gives biologists who study behavior access to the latest video-understanding models, streamlining behavioral analyses and enabling experiments that were previously out of reach, thereby accelerating discovery across fields.

## Methods

### Datasets

We evaluated FERAL across seven datasets spanning a range of taxa, behavioral contexts, and recording modalities. All datasets provided raw video data paired with frame-level behavioral annotations, enabling direct video-to-behavior mapping. Other open-source datasets containing only keypoint trajectories or bounding boxes without frame-level behavioral labels were excluded, as the current implementation focuses on frame-level classification. Training and test statistics, including the number of frames per split for each dataset, are reported in Table 2, and class distributions are given in Table 3. Note that for single-class classification, we include an “other” class that the model is trained to predict when no other relevant behaviors are present. For all reported class-averaged metrics, the “other” class is excluded

### CalMS21 (Mice social interactions)

The Caltech Mouse Social Interactions (CalMS21) dataset captures freely behaving mice engaged in resident–intruder assays [35]. Each recording includes tracked pose keypoints and frame-level labels for social behaviors such as attack, mount, and investigation. Following prior work, we evaluated performance using the mean average precision (mAP) metric on the same train–test split defined in the original challenge.

### MaBE (Multi-Agent Behavior Benchmark)

MaBE [36] is a large-scale multi-species benchmark designed for evaluating multi-agent behavior analysis. It includes recordings of mice, beetles, ants, and flies paired with expert-annotated behavioral categories. As only the beetle subset provides raw video aligned with human frame-level labels, we restricted our evaluation to this partition. Behavioral categories included locomotion, contact, and interaction states, annotated by trained ethologists. We report macro-averaged F1 scores following the evaluation protocol described in the original publication.

### PanAf500

PanAf500 [41] is a dataset of wild primate behavior, featuring both chimpanzees and gorillas recorded by camera traps across multiple African field sites within the Pan African Programme. The cameras operate during the day and at night, capturing a range of behaviors expressed by single individuals and groups under diverse weather and lighting conditions.

The original work introduces two datasets: PanAf20k, containing 20k clips with a single behavior label per clip, and PanAf500, consisting of 500 clips in which each individual ape is detected with a bounding box in every frame and assigned a behavior label. Because we wanted to evaluate FERAL on per-frame behavior classification, we used the smaller PanAf500 subset. For this dataset, we ignored the bounding box coordinates and used only the behavior labels, pooling behaviors across all bounding boxes in a frame into a single multi-label target (e.g., if two apes were labeled as sitting and one as climbing, the frame-level target would be sitting and climbing).

In our experiments, we retained the original training split (400 15s videos) and constructed a single held-out test set by combining the validation and test splits from the original paper (100 15s videos).

Because we significantly changed the objective for this dataset, we do not compare our performance to the results reported in the original paper. For PanAf500, the original work reports per-behavior accuracies by predicting a single class for individual behavior sequences for each detected animal, whereas we predict multiple classes for the whole frame at each frame of the video. Beyond the original setup, strong results on this dataset have also been obtained using self-supervised pretraining of a V-JEPA2 model [44]. However that approach follows the original work by first cropping individual animals before applying a separate classifier, while also adding an extra pretraining stage. In contrast, for this dataset we intentionally keep the pipeline simple and evaluate how well a model can perform without any intermediate detection or cropping steps.

### *C. elegans* locomotion

Animals were grown at 20°C on nematode growth media (NGM) plates seeded with *E.coli* OP50 bacteria [45]. Experiments were performed on young adult hermaphrodites (picked as L4s 14 – 16hrs before).

Animals are expressing two transgenes: one extrachromsomal array kyEx = pSM(F23H12.7p: :ReaChR::sl2::GFP) at 70ng/uL + pSM( myo3p::mCherry ) at 3ng/uL and one integrated transgene kyls= = pSM(ser-4p::flp, sto3-p::frt::HisCl1::sl2::mCherry, ges-1p::nls-GFP). The animals were not exposed to all-trans-retinol nor histamine during the course of the recordings.

Animals were recorded while performing an off-food foraging assay [46, 47], using a plastic ring (6mm Clear Mylar Stencil Sheets) instead of *CuCl*_2_ as a boundary to contain the animals. Preconditioning plates were made the night before by seeding NGM plates with a thin uniform OP50 lawn 16hrs prior to the start of the assay. 45 minutes prior to the start of the assay, a 1 in × 1 in plastic ring was placed on the preconditioning plate as a boundary and 20 young adult worms were picked onto the lawn. 5 minutes prior to the start of the assay, 8–11 animals were transferred to an unseeded NGM plate to clean off excess food, then transferred to the assay plate, an unseeded 10cm NGM plate with a plastic ring (2 in × 2 in) boundary to keep animals within the recording field of view. Behavior was recorded for 45 min using a Basler ace acA5472–17 μm USB 3.0 Monochrome Camera at 6 fps and 3,648px × 3,648px FOV.

Animals were tracked using custom python software which segments the worms from background using thresholding and generates tracks of the centroid of segmented worms using intersection over union. This results in tracks including centroid and mask of the worm.

Behavior was classified heuristically using rules adapted from [48, 49] implemented in custom python software. For each frame, it was determined if the animal was self-intersecting by using the ratio of the area: perimeter ratio of the outermost contour of the mask (ratio>3.3). For non self-intersecting frames, masks were skeletonized to get the midline of the worm and aligned in the same direction by minimizing the distance between midline points in adjacent frames. The head-tail vector was taken from the first and last midpoints of the worm.

Velocity was calculated by taking the difference between centroids 4 frames (=0.67s) apart and dividing by dt = 0.67s. Head versus tail was assigned as the direction along the head-tail axis the animal moves in more often (the animal moves towards the direction of its head more often than the direction of its tail). Speed was calculated by taking the absolute magnitude of the velocity. Speed was signed by the direction of the velocity relative to the direction of the head-tail vector.

Turning was classified as frames in which either a) both i) midpoint-tail vector (the vector connecting the midpoint and the tail of the worm) was greater in length than the midpoint-head vector (the vector connecting the midpoint to the head of the worm) and ii) the angle between the midpoint-tail vector and midpoint-head vector was less than 45 degrees or b) when the animal was self-intersecting.

Pausing was classified as frames for which speed < 50 μm/s for at least 0.5s and for which the animal was not turning.

Forwards was classified as speed greater than 0 μm/s and not turning or pausing.

Reversal was classified as speed less than 0 μm/s and not turning or pausing.

### Collective behavior and adult-larva interactions in clonal raider ant (*Ooceraea biroi*)

Stock colonies of *Ooceraea biroi* were maintained in constant light at 25°C in Tupperware containers (40 × 26 cm) with a 2 cm thick plaster of Paris floor. Colonies were fed with frozen fire ant (*Solenopsis invicta*) brood following the lab’s regular feeding schedule (3 times per week) and cleaned and watered once per week, as needed.

For behavior experiments, adult ants and fourth-instar larvae from the same stock colony (clonal line B genetic background) were collected and housed in 5 cm Petri dishes lined with a plaster of Paris floor and kept at 25°C. Behavioral assays were performed in a custom-built acrylic chamber with transparent sides and plaster of Paris floor. In each assay, an adult ant was allowed to settle in the chamber for 30 minutes before a larva was introduced. Videos were recorded from the side at 10 frames per second through a FLIR blackfly camera (BFS-U3–50S5C-C) and lens (Computar, MLM3X-MP), using Spinnaker Software Development Kit, at a pixel resolution of 2448px × 2048px. Recordings were collected across three consecutive days.

Behavioral annotations of adult ants and larvae were performed manually using ELAN [50]. We scored adult self-grooming and adult allogrooming of larvae, and recorded the start and end times and duration of these behaviors (in milliseconds).

For the colony tracking experiment, groups of 100 adults and 100 larvae were randomly subsampled from stock colonies and established in Petri dishes (90 × 20mm) lined with a humidified plaster of Paris base. Sub-colonies were allowed to acclimate for four days. Sub-colonies were then continually video recorded at 0.1 fps for 30 days under constant illumination at 25°C. Approximately every two days colonies were fed with frozen *Solenopsis invicta* brood and were cleaned approximately every two days.

### Zebras recordings from drones

Herds of Grevy’s zebra were filmed at Mpala Conservancy in Laikipia, Kenya in 2017 and 2018 using DJI Phantom 4 Pro Drones (DJI, Shenzhen, China). Drone import and operations were authorized by the Kenya Civil Aviation Authority (KCAA) and carried out by a licensed pilot assisted by an observer who maintained visual contact with the drone and a ground observer who maintained situational awareness. During filming, the drones were positioned directly above the group at a height of approximately 85 m above ground level (AGL), and followed the group’s movements. To achieve continuous observations longer than the drone’s battery duration, two drones were flown in a relay: when one drone’s battery became depleted, a second drone was positioned 10 m above the first one. The first drone was then recalled to the launch point and the second drone lowered down 10 m to continue following the group. If animals seemed disturbed by the drone (e.g. running away) or moved too far from the launch point, the observation was terminated. When groups were calm and remained in range, observations spanned 3 drone flights (approximately 45–50 minutes). During the first two flights, groups were filmed in an undisturbed state. During the third flight, researchers approached the group on foot to elicit a detection and flight response, during which the drone followed the group until they ran out of range. All fieldwork in Kenya was conducted with the permission of the National Commission for Science, Technology and Innovation and in affiliation with the Kenya Wildlife Service. Data collection protocols were reviewed and approved by Ethikrat, the independent ethics council of the Max Planck Society.

Drone recordings were initially captured at 4K resolution and 60 fps, but were downsampled to 30 fps prior to further processing. A multi-stage pipeline was applied to generate continuous movement trajectories for all animals in the recordings [40]. Recordings were then cropped to generate individual videos of a small square area (160 × 160 px – 210 × 210 px) centered on each animal. Forty-five individual videos from four observations were then manually annotated in BORIS [31] to identify bouts of vigilance behavior, defined as the animal standing still with its head raised, and periods when the animal was out of sight, for example due to passing under occluding vegetation. All other video frames were uncategorized.

### Model Architecture

FERAL fine-tunes a state-of-the-art video-understanding backbone to perform frame-level behavioral classification across predefined categories. The model outputs class probabilities for every frame. The complete default configuration used for all reported dataset, including all hyperparameters, is available on GitHub^1^

#### Backbone evaluation and selection

We systematically evaluated several recent video-understanding architectures as potential encoders for FERAL, focusing on their balance of accuracy, computational efficiency, and ease of deployment.

##### InternVideo2.

InternVideo2 is a 1-billion-parameter transformer model trained in multiple pretraining stages [51]. Despite strong representational capacity, fine-tuning proved prohibitively expensive, requiring up to eight hours for a full run on the CalMS21 dataset using four H100 GPUs. Furthermore, the codebase depended on numerous bespoke modules, complicating installation and development. While performance was promising, these practical limitations rendered InternVideo2 unsuitable for a user-friendly behavioral analysis framework.

##### SmallVLM2.

We next assessed SmallVLM2, a multimodal video–language model available in configurations ranging from 256M to 2.2B parameters [52]. Although integration via Hugging Face Transformers greatly simplified deployment, performance lagged behind state-of-the-art methods. The model processed frames largely in isolation, aggregating temporal information only in a shallow pooling layer. This architectural constraint, compounded by the predominance of non-video modalities during pretraining, limited its ability to capture long-range motion dynamics. Even with extensive regularization (including partial freezing, data augmentations, and label smoothing) SmallVLM2 exhibited overfitting on small datasets and failed to generalize on internal benchmarks.

##### V-JEPA2.

Based on these observations, we adopted V-JEPA2 as the encoder [29]. Unlike video–language models, V-JEPA2 is a dedicated video foundation model trained self-supervised on over one million hours of unlabeled video using a masked prediction objective. We employed the smallest available configuration (330M parameters) finetuned on the Diving48 dataset[53], which offers a favorable trade-off between performance and computational cost. Fine-tuning enables FERAL to align pretrained spatiotemporal representations with the specific requirements of behavioral segmentation, achieving great performance across benchmarks with modest data volumes and standard GPU resources.

#### Classification head

To convert spatiotemporal embeddings from the backbone into frame-level behavioral predictions, we designed a lightweight classification head that aggregates contextual information and outputs per-frame logits across behavioral classes.

Each input video chunk is represented as a sequence of thousands of spatiotemporal tokens. These tokens are first processed through transformer layers of the V-JEPA2 encoder[54] that enrich each token with contextual information from the entire sequence. To map this long sequence onto the temporal resolution of the input video, we employ an attention-based pooling module. Specifically, a set of 64 learnable query embeddings cross-attend to the encoder outputs, extracting features that correspond to individual frames.

The resulting pooled embeddings are flattened and passed through a Batch Normalization layer [55], which stabilizes training and controls feature variance, followed by a dropout layer (*p* = 0*.*5) [56] to reduce overfitting. A final linear projection maps the normalized embeddings to class logits for each frame.

#### Loss function

FERAL employs different loss functions for single-label and multi-label classification. For single label, FERAL is trained using a cross-entropy loss computed at the frame level. To improve generalization and mitigate overconfidence, we apply label smoothing with a factor of 0.1, which encourages the model to distribute probability mass across semantically related classes rather than assigning absolute certainty to a single label.

Because behavioral datasets often exhibit pronounced class imbalance, particularly between dominant “background” states and rare but biologically meaningful actions, we incorporate class-specific weighting into the loss. We found that scaling weights by the square root of the inverse class frequency yielded better performance amplifying the contribution of underrepresented behaviors without overcompensating, compared to using inverse-frequency weighting.

In the multi-label setting, FERAL is trained with binary cross-entropy loss, weighting each class by $\sqrt{\frac{N\;\text{positives}}{N\;\text{negatives}}}$ to emphasize rare classes.

We additionally evaluated focal loss, which dynamically down-weights easy examples to focus learning on difficult cases, but found no consistent improvement across benchmarks.

#### Chunking strategy

Transformer-based video encoders compute pairwise attention across all spatiotemporal tokens, causing computational complexity to scale quadratically with the number of input tokens. As a result, processing entire behavioral recordings end-to-end is infeasible. Consequently FERAL divides each video into overlapping segments, or *chunks*, of fixed length before processing. Each chunk comprises 64 consecutive frames, resized to 256 × 256 pixels.

To capture fine-grained behavioral dynamics, consecutive chunks overlap by 50% (i.e., stride = 32 frames). This design ensures that short behaviors spanning chunk boundaries remain fully visible within at least one receptive field. During training, overlapping windows also increase the effective number of training samples further improving quality.

During inference, as the model outputs per-frame predictions for each chunk, we employ a unified post-processing pipeline to gather the final frame-level predictions. If a frame received multiple predictions, we averaged the corresponding class probabilities. For frames without a direct prediction, we linearly interpolated probabilities from the two nearest frames with predictions. This approach effectively ensembles model’s own local predictions.

**Extended Figure 1.**
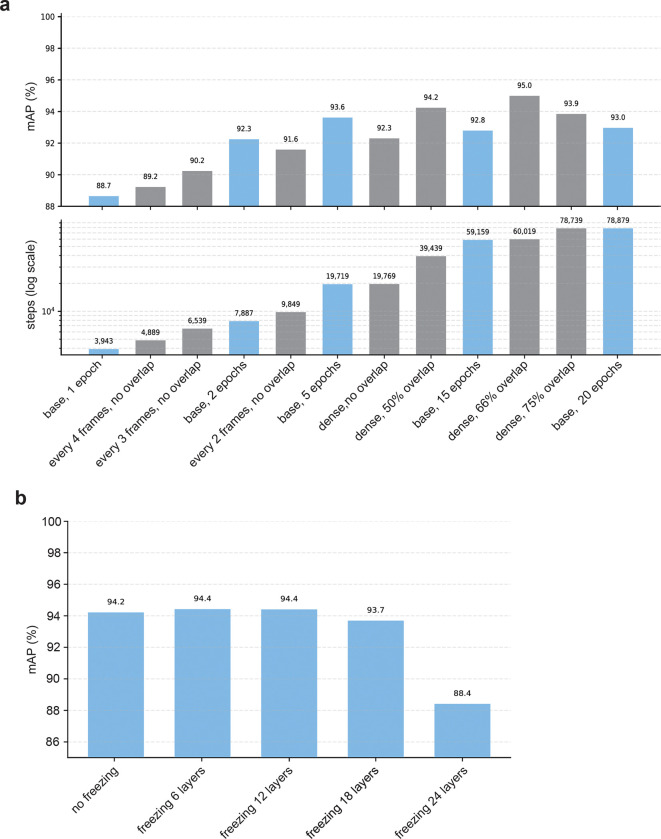
Effect of temporal sampling and backbone freezing on FERAL performance. **(a)** Model quality as a function of number of training steps and overlapping-chunk configuration on the CalMS21 dataset. **(b)** Evaluation of layer-freezing strategies during fine-tuning

We benchmarked multiple configurations varying both stride and sampling rate (Extended Figure 1) and found that sampling every frame with 50% overlap yielded the best balance between accuracy and training speed.

#### Augmentations

To improve generalization across diverse lighting conditions, species, and recording setups, FERAL employs a combination of video and label-space augmentations. For visual augmentation, we adopted *TrivialAugment* [57], which randomly samples from a set of standard image transformations (e.g., brightness, contrast, rotation, color jitter) and applies them at varying strengths. The same augmentation was applied consistently across all frames within a video, preserving temporal coherence while introducing diversity across video samples.

In addition, we applied *MixUp* regularization at the batch level [58]. Each augmented sample was formed as a convex combination of two videos, X and $\tilde{X}$, and their corresponding label sequences, y and $\tilde{y}$:

$$X_{\text{new}}=\alpha X+(1-\alpha )\tilde{X},\;y_{\text{new}}=\alpha y+(1-\alpha )\tilde{y},$$

where α∼Beta(λ,λ). Because FERAL operates at frame resolution, label mixing was performed element-wise across the temporal dimension. This strategy helps to mitigate overfitting on small datasets.

#### Training

We use the Adam optimizer [59] with a relatively strong weight decay (0*.*1) to counteract overfitting given the modest size of behavioral datasets. The learning rate follows a schedule consisting of a linear warm-up for the first 20% of iterations, followed by cosine decay. Models are trained for 10 epochs, which we found sufficient for convergence across benchmarks.

Unlike approaches such as VideoPrism, which freeze the backbone and train only shallow classifiers, FERAL fine-tunes the last 12 out of 24 transformer layers in VJEPA2, aligning high-level spatiotemporal embeddings with behavioral structure.

To further support generalization and out-of-distribution performance, we allow users to enable an optional weight-averaging feature. When activated, FERAL will maintain an exponential moving average (EMA) $\theta_{EMA}$ of the model weights θ, which at step t is computed as [60]:

$$\theta_{t+1}^{EMA}=\beta \theta_{t}^{EMA}+(1-\beta )\theta_{t},$$

with β=0.999. During evaluation, both the standard and EMA-weighted models are assessed and users can select the best performing option for their application. In our reported experiments, we do not report metrics from EMA checkpoints, as some of the datasets are relatively small and we preferred to keep the evaluation protocol simple rather than risk over-optimizing on them.

### Experiments

#### Data efficiency

We tested FERAL’s data efficiency by training on smaller CalMS21 subsets and measuring performance drop versus the full-data baseline, while keeping the test set and all hyperparameters the same. We implemented two complementary subsampling schemes:

##### (1) Video-level subsampling.

We randomly sampled subsets of training videos at 50% (mAP 92.0%) and 25% (mAP 93.0%) and trained FERAL on these reduced sets. Because individual recordings vary in length and behavioral composition, smaller subsets (<25%) produced high variance across runs: some samples didn’t have all the classes present and the total number of frames varied substantially. We therefore do not report results below 25%.

##### (2) Chunk-level subsampling.

To probe sample efficiency under more balanced class distributions, we first processed the full training set into ready-to-train chunks and then randomly subsampled from them. This design mitigates class imbalance and enabled evaluation on much smaller training sets, down to 1% of the original data.

Since expanding datasets typically involves annotating additional videos, video-level subsampling best reflects realistic scaling. However, only chunk-level subsampling allows evaluation under extreme reductions without manual video selection. Under both regimes, training on 25% of the data still exceeded the prior SOTA, and other reduced-data settings maintained strong performance. These results indicate that FERAL’s foundation-model backbone and fine-tuning strategy confer substantial sample efficiency, enabling high performance with limited labeled footage. This is especially valuable in behavioral research, where manual annotation is expensive and time-consuming

### Chunking strategies

We systematically benchmarked chunking strategies to balance computational efficiency and quality. Each configuration is defined by two parameters:

- **Frame stride**: the interval between frames within a chunk (e.g., every frame, every second frame). Larger strides expand the effective temporal window but reduce temporal resolution.
- **Chunk overlap**: the proportion of frames shared between consecutive chunks (e.g., 0%, 50%, 75%). At 0% each frame in the video appears in only one chunk, at 50% in two, at 66% in three, etc. Greater overlap increases computation but improves contextual continuity and yields multiple predictions per frame that can be ensembled to enhance quality.

Performance improved monotonically as stride decreased, with the best results at stride 1 (sampling every frame; labeled as “dense” on Extended Figure 1a). Adding overlap yielded substantial quality gains, with 66% overlap producing the highest mean average precision (95.0%). Notably, even sparse settings (0% overlap, sampling every fourth frame) exceeded the competition baseline while requiring 8 times less steps than our default configuration.

To test whether improvements were simply due to more training steps, we matched the total number of optimization steps by training the base configuration (50% overlap, dense sampling; blue bars on Extended Figure 1a) for fewer or more epochs. Dense configuration were clearly superior to sampling every other frame, and moderate overlap was beneficial, although returns diminished at higher overlaps. We therefore adopted 50% overlap with full-frame sampling as the default.

### Freezing strategies

To evaluate the impact of layer freezing on performance, we incrementally froze six-layer blocks of the 24-layer V-JEPA2 encoder, proceeding from the input forward. Partial freezing (up to 12 layers) slightly improved test quality, consistent with mild regularization. In contrast, freezing the entire encoder markedly reduced performance (mAP of 88.4%). These results indicate that fine-tuning at least the final layers is necessary to align pretrained features with behavioral segmentation tasks (Extended Figure 1b).

### Metrics

We report mean average precision (mAP) and macro-averaged F1, precision, and recall. mAP is computed from calibrated probabilities, excluding the “other” category from averaging. For multi-label tasks (e.g., MaBE), results are reported using a fixed threshold of 0.85 for all classes. You can find the performance metrics of FERAL across all benchmark datasets using the same default configuration in Table 1.

### Metric rationale

Accuracy was not used, as behavioral datasets typically exhibit substantial class imbalance. Datasets often include a frequent “other” class, while biologically interesting events are relatively rare (often *<* 1% of frames). In such settings, accuracy overestimates performance by rewarding majority-class predictions. Instead, we report mAP, which offers a more sensitive measure of per-class discrimination across thresholds, while macro F1, precision, and recall provide a complementary view of performance. mAP is computed from calibrated probabilities over the full dataset, whereas the F1 score is computed after thresholding continuous model outputs into discrete predictions. For all reported averages, the “other” class was excluded, as it denotes the absence of annotated behaviors rather than a discrete category.

### Model selection

For all runs, we train the model on the full training dataset and evaluate on the test dataset afterward. We observed that splitting the available non-test data into training and validation sets and selecting the best-performing checkpoint across epochs to evaluate on the test set led to highly variable results, due to the small size of some datasets. Therefore, we chose this setup, accepting that mild overfitting on some datasets is preferable to undertraining on others.

### Run tracking

Each training run automatically logs all metrics to Weights & Biases (W&B), providing users with real-time visualization and reproducibility. Logged outputs include low-level training diagnostics (e.g., loss, learning rate), per-class average precision at both chunk and frame levels, aggregated mAP, and qualitative ethograms after each validation epoch. Each run generates a unique dashboard URL, enabling easy sharing and comparison.

### Engineering and deployment

#### Video preprocessing and seekability.

Training requires fast access to arbitrary frames in the video. Many common codecs support only sequential reads and do not allow frame-level seeking, so re-encoding is often necessary. Pre-resizing can also help with HD videos, as decoding large videos may be too slow compared with GPU computation speed. FERAL includes a cross-platform utility that converts input videos to seekable formats and resizes them to 256×256. If source videos are not high resolution and are already seekable, users may skip re-encoding; resizing will then be performed dynamically during training

#### Hugging Face integration.

To ensure modularity and ease of extension, FERAL leverages the Hugging Face transformers library [61] for model management. This abstraction simplifies switching between backbones and streamlines installation compared to earlier architectures (e.g., InternVideo2), which relied on bespoke code and complex dependencies.

#### Training visualization and monitoring.

FERAL integrates with Weights & Biases (W&B) for experiment tracking, allowing users to monitor training, validation and test performance. Logs are also stored in the cloud, enabling training across multiple servers while maintaining a unified analysis interface. Users can connect their own W&B accounts or use a public workspace provided by FERAL.

#### Deployment and compute requirements.

FERAL requires 24 GB of VRAM for training, which fits on high-end consumer GPUs. All reported experiments were conducted on NVIDIA H100 and L40s GPUs in a university cluster. For users without access to high-performance computing, FERAL provides step-by-step deployment guides for GPU cloud platforms (e.g., RunPod) and a Google Colab notebook, enabling full training runs at low cost without complex setup. All necessary dependencies, including CUDA and PyTorch [62], are pre-installed on these instances, allowing users to begin training within minutes.

## Extended Data

**Table 1:** Performance metrics across all evaluated datasets. All experiments were conducted using the default configuration.

Dataset	mAP	F1	Precision	Recall

CalMS21	0.945	0.893	0.89	0.895
Ant colonies	0.978	0.928	0.936	0.920
C. elegans	0.932	0.863	0.820	0.921
Adult-larva ants	0.925	0.813	0.728	0.930
MaBE	0.970	0.920	0.951	0.894
Zebras	0.853	0.785	0.809	0.768
PanAf500	0.657	0.578	0.667	0.527

**Table 2:** Summary of datasets used in our experiments

Dataset	Classes	Train videos	Train frames	Test frames	Test videos	Multilabel

CalMS21	4	70	507738	262107	19	False
Ant colonies	2	7	126000	36000	2	False
C. elegans	5	29	92560	35674	8	False
Adult-larva ants	3	7	126000	54000	3	False
MaBE	6	897	807300	202500	225	True
Zebras	3	35	2487594	424550	9	False
PanAf500	9	400	143959	35998	100	True

**Table 3:** Class frequency distribution (percentage of labeled frames across train and test splits) for each dataset.

Dataset	Class distribution (%)

CalMS21	*attack*: 3.5%, *investigate*: 27.0%, *mount*: 7.9%, *other*: 61.7%
Ant colonies	*other*: 91.7%, *raiding*: 8.3%
C. elegans	*other*: 0.1%, *forward*: 84.6%, *reverse*: 7.3%, *turn*: 2.3%, *pause*: 5.8%
Adult-larva ants	*other*: 88.9%, *self* : 1.4%, *larvae*: 9.7%
MaBE	*behavior* 1: 21.1%, *behavior 2*: 15.5%, *behavior 3*: 21.3%, *behavior 4*: 7.8%, *behavior 5*: 24.6%, *behavior 6*: 9.6%
Zebras	*other*: 78.1%, *out of sight*: 2.4%, *vigilant*: 19.5%
PanAf500	*camera interaction*: 1.1%, *climbing down*: 0.8%, *climbing up*: 2.2%, *hanging*: 4.8%, *running*: 1.0%, *sitting*: 32.4%, *sitting on back*: 1.4%, *standing*: 21.6%, *walking*: 27.1%

## Data Availability

All source code, training configurations, and documentation for FERAL are publicly available at www.getferal.ai and https://github.com/Skovorp/feral. The website and the repository include example datasets, preprocessing utilities, and instructions for local and cloud-based deployment. Data and Supplementary Videos 1–4 used in this publication are available at the GitHub Data Repository (https://github.com/Skovorp/feral_share_data/tree/main)

## Footnotes

1
https://github.com/Skovorp/feral/blob/main/configs/default_vjepa.yaml
